# Temporal trend and factors associated with spatial distribution of congenital syphilis in Brazil: An ecological study

**DOI:** 10.3389/fped.2023.1109271

**Published:** 2023-03-22

**Authors:** Janmilli da Costa Dantas, Cristiane da Silva Ramos Marinho, Yago Tavares Pinheiro, Maria Ângela Fernandes Ferreira, Richardson Augusto Rosendo da Silva

**Affiliations:** ^1^ Graduate Program in Collective Health, Health Science Center, Federal University of Rio Grande Do Norte, Natal, Brazil; ^2^ Graduate Program in Collective Health, Faculty of Health Sciences of Trairi, Federal University of Rio Grande Do Norte, Santa Cruz, Brazil

**Keywords:** *Treponema Pallidum*, prenatal care, infectious diseases, spatial analysis, space time clustering

## Abstract

**Objective:**

The study aimed to analyze the temporal trend of congenital syphilis in Brazil in the period from 2008 to 2018 and its spatial distribution in the Immediate Regions of Urban Articulation, and to identify spatial correlations with socioeconomic factors and prenatal care.

**Methods:**

Spatial correlations between the incidence of congenital syphilis and socioeconomic conditions and access to prenatal care were assessed. This ecological study conducted a time series analysis in Brazil and spatial analysis in 482 Immediate Regions of Urban Articulation. Cases of congenital syphilis reported in the Notifiable Diseases Information System and the Live Birth Information System from January 1, 2008, to December 31, 2018 were included. Socioeconomic conditions (percentage of individuals with inadequate water supply and sanitation) were extracted from the 2010 census, whereas the Live Birth Information System provided data on access to prenatal care (percentage of live births with 1–3 prenatal care appointments). The Joinpoint Regression software performed the temporal trend analysis, while the GeoDa software assessed territorial clusters using the Moran’s *I* and Local Spatial Association Indicator.

**Results:**

The incidence of congenital syphilis showed an upward trend (annual percent change 1 = 26.96; 95% CI: 18.2–36.3; annual percent change 2 = 10.25; 95% CI: 2.7–28.4) and was unevenly distributed across Immediate Regions of Urban Articulation in Brazil (Moran’s *I* = 0.264, *p* ≤ 0.05). It also presented a direct spatial correlation with the percentage of individuals with inadequate water supply and sanitation (Moran’s *I* = 0.02, *p* ≤ 0.05) and the percentage of live births with 1–3 prenatal care appointments (Moran’s *I* = 0.03, *p* ≤ 0.05).

**Conclusion:**

Agrowth trend of congenital syphilis in Brazil was observed between 2008 and 2018. Moreover, inequalities in socioeconomic conditions and access to prenatal care influenced the spatial distribution of this disease.

## Introduction

Pregnant women infected with *Treponema pallidum* and not diagnosed and treated during prenatal care may transmit syphilis to the fetus, leading to congenital syphilis (CS) (1, 2). Adverse events from this infection (e.g., abortion, stillbirth, premature birth, neonatal death, and early or late congenital manifestations) also impact maternal and neonatal health (3).

In 2016, 355,000 adverse birth outcomes due to CS were reported in the world, including 143,000 fetal deaths, 61,000 neonatal deaths, and 41,000 premature births with low birth weight newborns. In this same year, the highest and lowest incidences were observed, respectively, in the African (12 cases per 1,000 live births) and European (0.2 cases per 1,000 live births) continents. The worldwide incidence of CS decreased from 5.4 to 4.7 cases per 1,000 live births between 2012 and 2016, except in the American and the Eastern Mediterranean Regions. In this period, CS increased from 3.07 to 3.19 cases per 1,000 live births in the Americas, with Brazil reporting 85% of cases in Latin America (4). Also, Brazil recorded 260,596 cases of CS from 1998 to 2020; 22,065 cases were reported only in 2020, resulting in an incidence of 7.7 cases per 1,000 live births (5).

In 2016, the World Health Organization (WHO) developed a program to reduce the incidence of CS to 0.5 or few cases per 1,000 live births by 2030 in 80% of countries. In 2017, eleven countries or territories obtained validation, according to criteria established by the World Health Organization with regard to the elimination of mother-to-child transmission of HIV and/or syphilis (6). However, controlling CS requires data on temporal evolution, spatial distribution, and factors associated with disease spread. These data may be used for evaluation, planning, decision-making, management, and development of effective health policies (7).

Temporal trend, spatial distribution, and factors associated with CS are commonly evaluated in states or cities, but researchers rarely consider the entire Brazilian territory. In addition, the analysis of CS correlating the geographic location with socioeconomic conditions and access to prenatal care may contribute to disease control.

The increase in CS is influenced by several factors, such as the socioeconomic characteristics of the population and access to health services. Research points out that the high GINI index (8), low education of women, disadvantaged social strata (9, 10) and low number of prenatal consultations (10) hinder the control of CS.

In this scenario, this study aimed to analyze the temporal trend of congenital syphilis in Brazil in the period from 2008 to 2018 and its spatial distribution in the Immediate Regions of Urban Articulation, and to identify spatial correlations with socioeconomic factors and prenatal care.

## Methods

This ecological study used secondary, aggregated, public-domain data from Brazilian cities. According to resolution 510/2016 of the Brazilian National Health Council, approval by an ethics committee for this type of research is unnecessary.

Brazil is the largest country in South America. Is divided into 5,565 cities, 27 states, including the Federal District, according to a regional model used by the Brazilian Institute of Geography and Statistics (IBGE), in 2010. Brazil can also be divided into five macro-regions: North, Northeast, Midwest, South, and Southeast (11). Moreover, the Urban Regional Division classifies the Brazilian territory into Extended (14 territories), Intermediate (161 territories), and Immediate Regions of Urban Articulation (RUAs) (482 territories). In this model, regions are contiguous, and each city is part of a territorial unit with limits unrestricted to state borders. Each region has a leading city that influences other cities by offering highly complex goods and services (12). Thus, our study used Brazil for time series analysis and Immediate RUAs for spatial analysis.

Data were collected between June and July 2021 from publicly accessible databases linked to the Ministry of Health of Brazil, the Department of Informatics of the Unified Health System (DATASUS), and the National Human Development Program.

The health information systems in Brazil present a temporal delay to make their data available for public access. During the data collection period, Information System for Notifiable Diseases (SINAN) provided data on reported cases of congenital syphilis up to December 2018. Thus, the extracted data referred to the period of 11 years, comprising the years from 2008 to 2018.

The incidence rate of congenital syphilis is the dependent variable of the study. This rate was calculated, for each year of the study, from the number of reported cases of congenital syphilis in the city in that year, divided by the number of live births in the city in the same period, and then multiplied by 1,000. In order to obtain the data of the variable Congenital Syphilis incidence rate, the number of reported cases of CS per residence was obtained from SINAN; and the number of live births was obtained from SINASC (Information System of Live Births).

The Brazilian Ministry of Health (13) adopts definition criteria for congenital syphilis notification. These criteria include every newborn, stillbirth, or abortion of a woman with untreated or inadequately treated syphilis; microbiological evidence of *Treponema Pallidum* infection in a nasal secretion sample or skin lesion, biopsy, or necropsy of an abortion, stillbirth, or child. It also defines CS as every child under 13 years of age with clinical, cerebrospinal fluid or radiological manifestation of congenital syphilis and a positive non-treponemal test (VDRL or RPR).

Several socioeconomic and health care-related variables that could have an influence on determining the incidence rate of congenital syphilis were calculated after extracting data from the information systems. Using the Geoda software, it was possible to detect the variables that showed significant spatial correlation with the incidence rate of congenital syphilis. Among the variables tested in the spatial correlation, two showed significant spatial correlation with the incidence rate of congenital syphilis: (1) the percentage of people in households with inadequate water supply and sanitary sewage system (obtained from the National Human Development Program), (2) percentage of live births with 1–3 prenatal consultations (obtained from SINASC). Thus, these two independent variables were adopted in the study because they were statistically significant in the spatialization of the data.

The inadequate water supply and sanitary sewerage system in a population is related to economic and social issues in municipalities with low HDI-M (14), thereby justifying, in this research, the use of the variable percentage of people in households with inadequate water supply and sanitary sewerage system as related to the socioeconomic conditions of a population. For adequate prenatal care, a minimum of six prenatal consultations is recommended (8). Accordingly, related to prenatal care, the research used the variable percentage of live births with 1–3 prenatal consultations.

Other variables were tested, but did not show significant spatial correlation with the incidence rate of CS: MDI-M, GINI index, illiteracy rate in women 15 years old and older, percentage of primary care coverage, percentage of live births with no prenatal consultations, percentage of live births with 4–6 prenatal consultations, percentage of live births with 7 or more prenatal consultations.

The incidence of CS and percentage of live births with 1–3 prenatal appointments were collected from 2008 to 2018, whereas data on socioeconomic conditions were obtained from the 2010 census.

Data on the incidence of CS were normalized for live births in the same period to minimize discrepancies in indicators due to different population sizes. The study variables are detailed in Table 1.

**Table 1:** Characteristics of dependent and independent variables used to assess the spatial distribution of congenital syphilis in immediate regions of urban articulation of Brazil from 2008 to 2018.

Variables	Concept	Data source
Incidence rate of congenital syphilis	Number of reported cases of congenital syphilis divided by the total number of live births in a determined location and time, multiplied by 1,000.	DATASUS (SINAN and SINASC) DATASUS → Health Information (Tabnet) → Epidemiological and Morbidity → Notifiable Diseases and Conditions—from 2007 onwards (SINAN) → Congenital Syphilis → Brazil by city → City of residence → Year of diagnosis → Period 2008 to 2018 http://tabnet.datasus.gov.br/cgi/tabcgi.exe?sinannet/cnv/sifilisbr.def Accessed on June 25, 2021 DATASUS → Tabnet → Vital Statistics → Born Alive from 1994 to 2019 → Born Alive → Born by residence of mother http://tabnet.datasus.gov.br/cgi/tabcgi.exe?sinasc/cnv/nvbr.def Accessed on June 25, 2021
Percentage of people in households with inadequate water supply and sanitary sewage system	People who live in houses where water supply comes from the general system and sewage is not collected by a sewage system or septic tank divided by the total population residing in permanent private houses, multiplied by 100. Only permanent private houses were considered.	PNUD Atlas Brazil → Collection → Library → Database → Demographic Census 2010 https://onedrive.live.com/?authkey=%21AbiV0mb1HeyuOxU&cid=124653557C0404EC&id=124653557C0404EC%2123017&parId=124653557C0404EC%2122899&action=locate Accessed on June 29, 2021
Percentage of live births with 1–3 prenatal care appointments	Number of live births with 1–3 prenatal care appointments in a determined location and time divided by the number of live births from the same location and period, multiplied by 100.	DATASUS → Tabnet → Vital Statistics → Live births from 1994 to 2019 → 1 to 3 Prenatal care appointments → Birth by residence of the mother, city, and year of birth → From 2008 to 2018 http://tabnet.datasus.gov.br/cgi/tabcgiexe?sinasc/cnv/nvbr.def Accessed on July 02, 2021

For the trend analysis, the incidence rate of congenital syphilis was used exclusively, adopting Brazil as the unit of analysis. The Joinpoint Regression software performed the temporal trend analysis by adjusting a series of trend lines and junction points on a logarithmic scale and testing annual trends. The annual percent change (APC) and 95% confidence intervals (95% CI) were also estimated and tested; the Monte Carlo permutation test assessed statistical significance (15).

The spatial analysis was performed with the variable “outcome” and the independent variables “percentage of people in households with inadequate water supply and sanitary sewerage system” and “percentage of live births with 1–3 prenatal consultations”, using as unit of analysis the *RUAs* of Brazil. The GeoDa software performed an exploratory analysis to assess the spatial distribution of variables. The Global Moran’s *I* and Local Indicators of Spatial Association (LISA) tests were applied. Global Moran’s *I* coefficients verified the spatial autocorrelation of variables. Results ranged from −1 (inverse correlation) to +1 (direct correlation); a value of 0 represented no correlation. Values of local Moran’s *I* of the 482 RUAs were submitted to the Moran’s Scatter Diagram with significance set at 5% (*p* ≤ 0.05). RUAs were classified into five categories: high-high (unit and neighbors had values higher than the average of the set), high-low (high value for the unit and low average values for neighbors), low-high (the unit had low value for a variable, whereas neighbors had values above the average of the set), low-low (values of the unit and neighbors were below the average of the set), and without significance (the unit had no defined association with neighbors) (16, 17).

## Results

The SINAN recorded 164,293 cases of CS in Brazil from 2008 to 2018; the lowest incidence was recorded in 2008 (1.18 cases per 1,000 live births) and the highest in 2017 (8.65 cases per 1,000 live births). Thus, the incidence of CS showed an upward trend, growing by over 450%.

**Figure 1:**
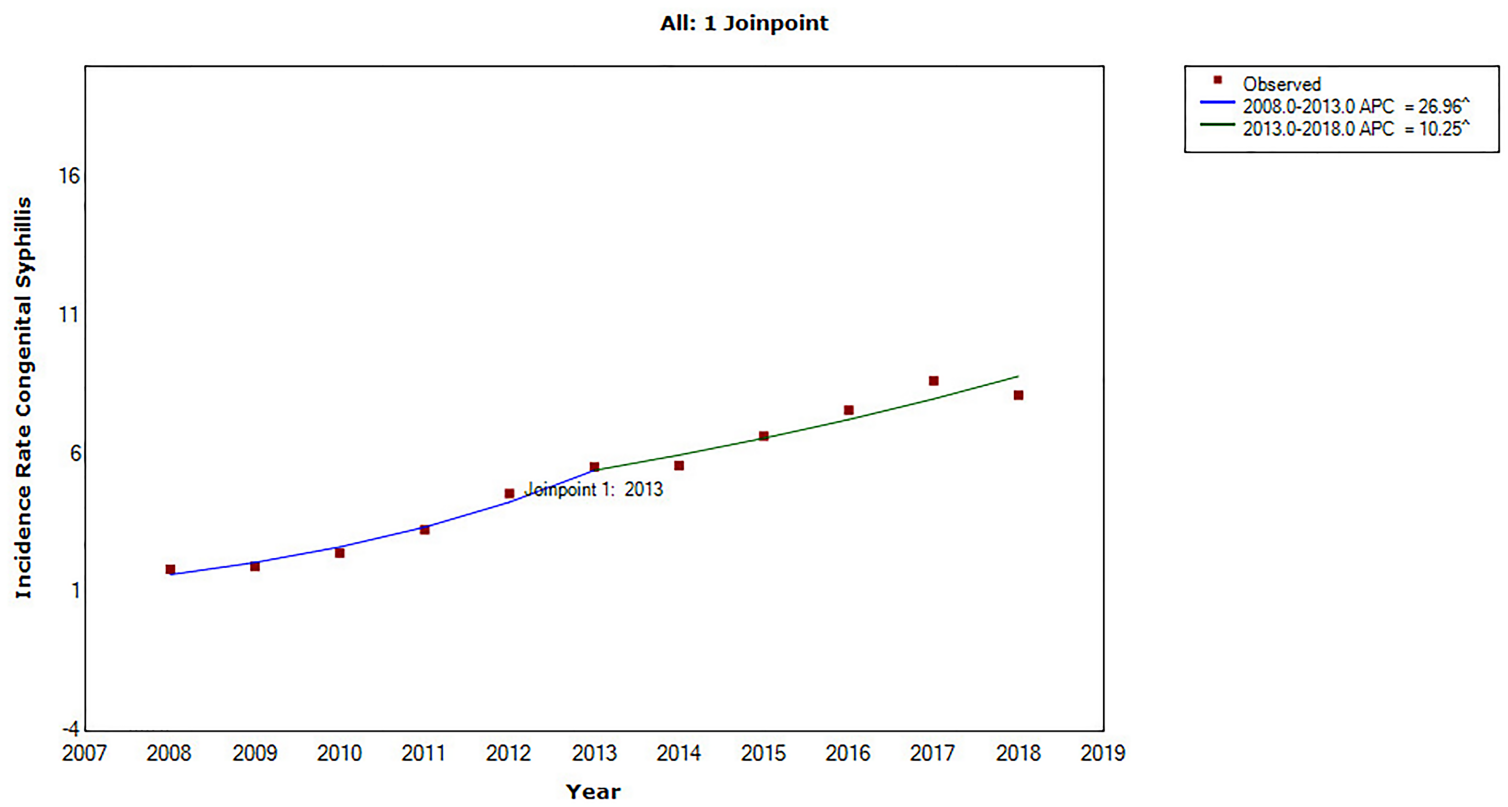
Time series of the incidence rate of congenital syphilis (dots) in Brazil from 2008 to 2018, according to the final selected model: 1 joinpoint. ^Indicates that the annual percent change (APC) is significantly different from zero at the alpha = 0.05 level.

An increasing trend was observed in the incidence of CS in Brazil (APC 1 = 26.96; 95% CI: 18.2–36.3; APC 2 = 10.25; 95% CI: 2.7–18.4) and an inflection point in 2013 (Figure 1 and Table 2).

**Table 2:** Incidence rate trend of congenital syphilis in Brazil and macro-regions, 2008–2018.

Variables	Brazil	Southeast	South	North East	North	Midwest
CS incidence rate 2008	1.83	1.92	1.15	1.88	2.40	1.48
CS incidence rate 2013	5.54	6.12	4.57	6.17	4.16	3.86
CS incidence rate 2018	8.13	8.87	8.36	8.40	6.41	5.59
Annual percent change (95% CI)	APC 1 = 26.96 (18.2–36.3) APC 2 = 10.25 (2.7–18.4)	APC 1 = 30.74 (16.8–46.4) APC 2 = 9.37 (−2.3 to 22.5)	APC 1 = 28.73 (25.3–32.3) APC 2 = −0.93 (−22.8 to 27.2)	APC 1 = 27.31 (21.6–33.2) APC 2 = 7.37 (2.6–12.4)	APC = 12.89 (9.8–16.1)	APC = 17.59 (13.7–21.7)
Joinpoints	1:2013	1:2013	1:2016	1:2013	0	0
Slope *p*-value	1) 0.000176 2) 0.015263	1) 0.00151 2) 0.100608	1) 0.000000 2) 0.930109	1) 0.000013 2) 0.008752	0.000004	0.000002
Trend	Increase	1) Increase 2) Stationarity	1) Increase 2) Stationarity	Increase	Increase	Increase

SINAN and SINASC (2008–2018).

**Figure 2:**
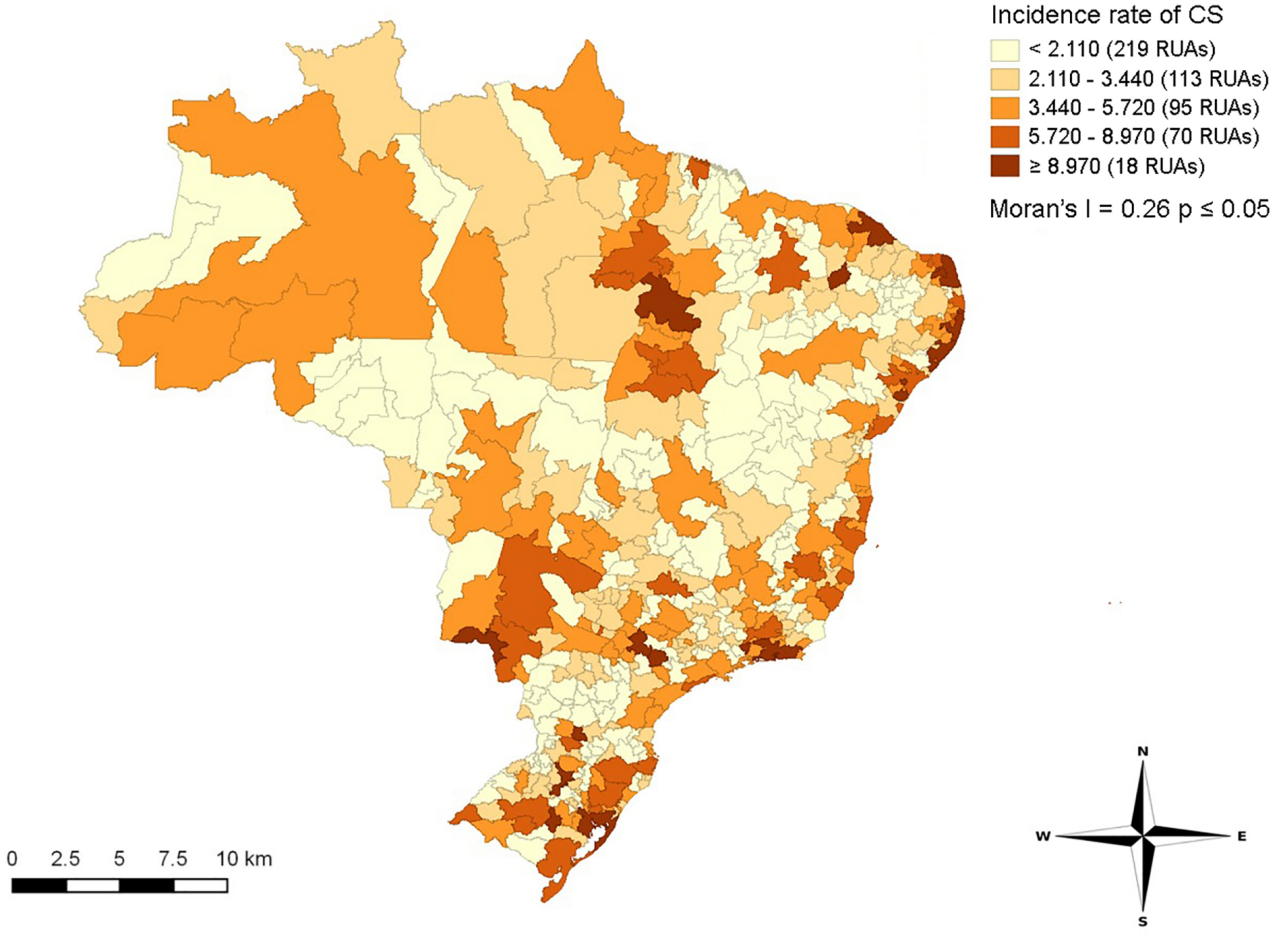
Spatial distribution of clusters (exploratory map) of the incidence rate of congenital syphilis, in immediate regions of urban articulation in Brazil, 2008–2018.

The clusters of the incidence rate of CS were presented unevenly in the RUAs of Brazil (Moran’s *I* = 0.26; *p* ≤ 0.05), between 2008 and 2018, as shown in Figure 2. Coastal areas in the Northeast region and Mato Grosso do Sul, Rio Grande do Sul, and Rio de Janeiro states presented the highest incidences of CS. Moreover, clusters in RUAs in the five macro-regions of the country were found, with the incidence of CS ranging from 3.44 to 5.72 cases per 1,000 live births. Clusters in RUAs presenting the lowest incidences of CS were also distributed in the Brazilian macro-regions, covering the central area of the Northeast and part of the Midwest, mainly the state of Mato Grosso.

**Figure 3:**
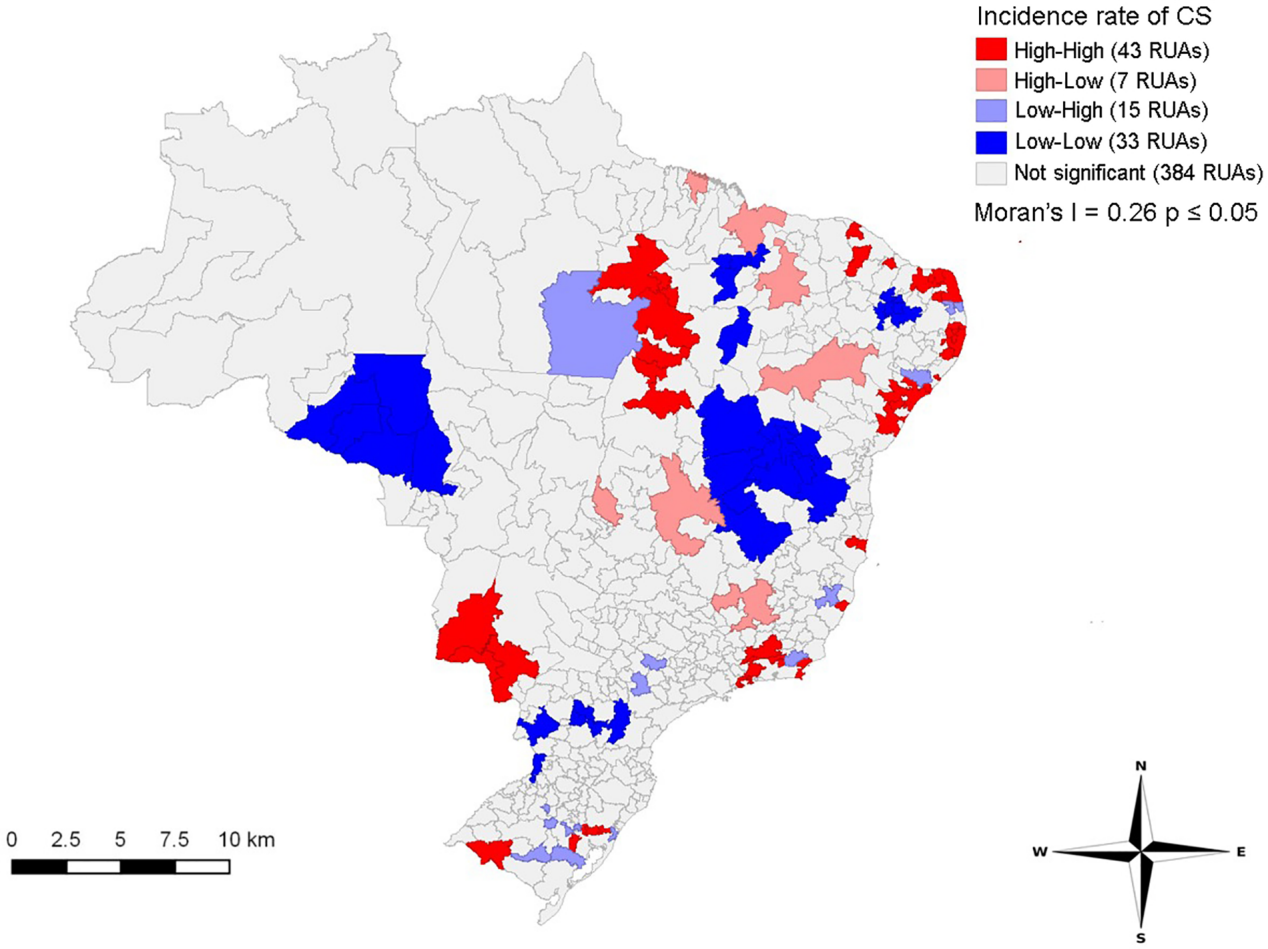
Spatial distribution of clusters (moran map) of the incidence rate of congenital syphilis in immediate regions of urban articulation in Brazil, 2008–2018.

Figure 3 shows clusters classified as high-high for the incidence rate of CS in coastal areas of the Northeast and small areas in the Southeast, South, and part of the Midwest. On the other hand, the central areas of the Northeast region and Tocantins, Rondônia, and Mato Grosso states presented low-low clusters for the incidence rate of CS.

**Figure 4:**
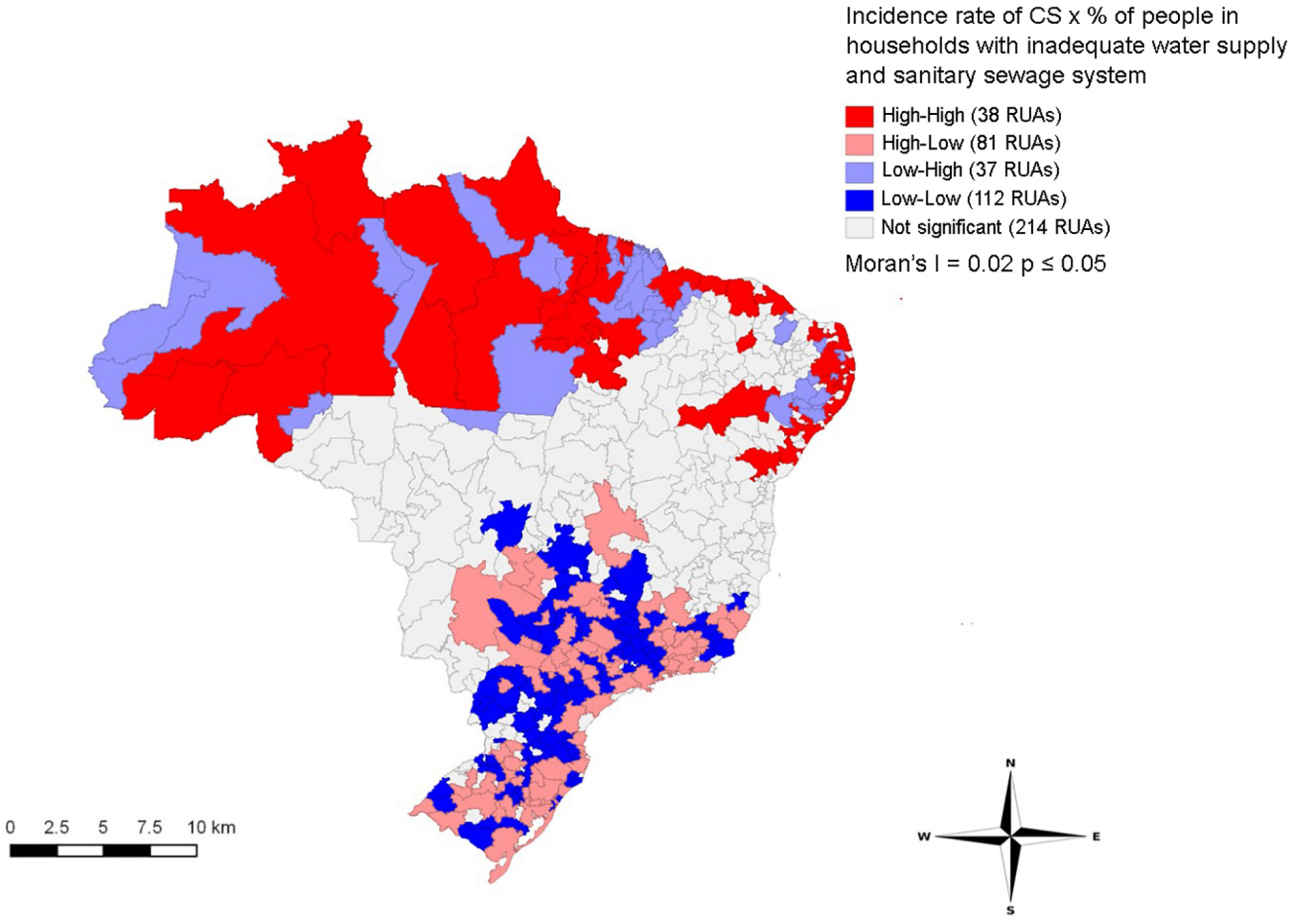
Distribution of LISA bivariate spatial correlation clusters of congenital syphilis incidence rate with the socioeconomic indicator in immediate regions of urban articulation in Brazil, 2008–2018.

Figure 4 shows direct spatial correlations between the incidence rate of CS and the percentage of individuals with inadequate water supply and sanitation (Moran’s *I* = 0.02, *p* ≤ 0.05). Clusters in RUAs from coastal areas of Northeast and North regions presented a high incidence rate of CS and percentage of individuals with inadequate water supply and sanitation. In contrast, the South, Southeast and small areas of the Midwest showed clusters in IRUAs with low percentages of individuals with inadequate water supply and sanitary sewage system and low incidence rates of congenital syphilis. The data reflect the influence of socioeconomic conditions in specific areas on the incidence of congenital syphilis, with areas with disadvantaged socioeconomic conditions being more affected by the incidence of the illness.

**Figure 5:**
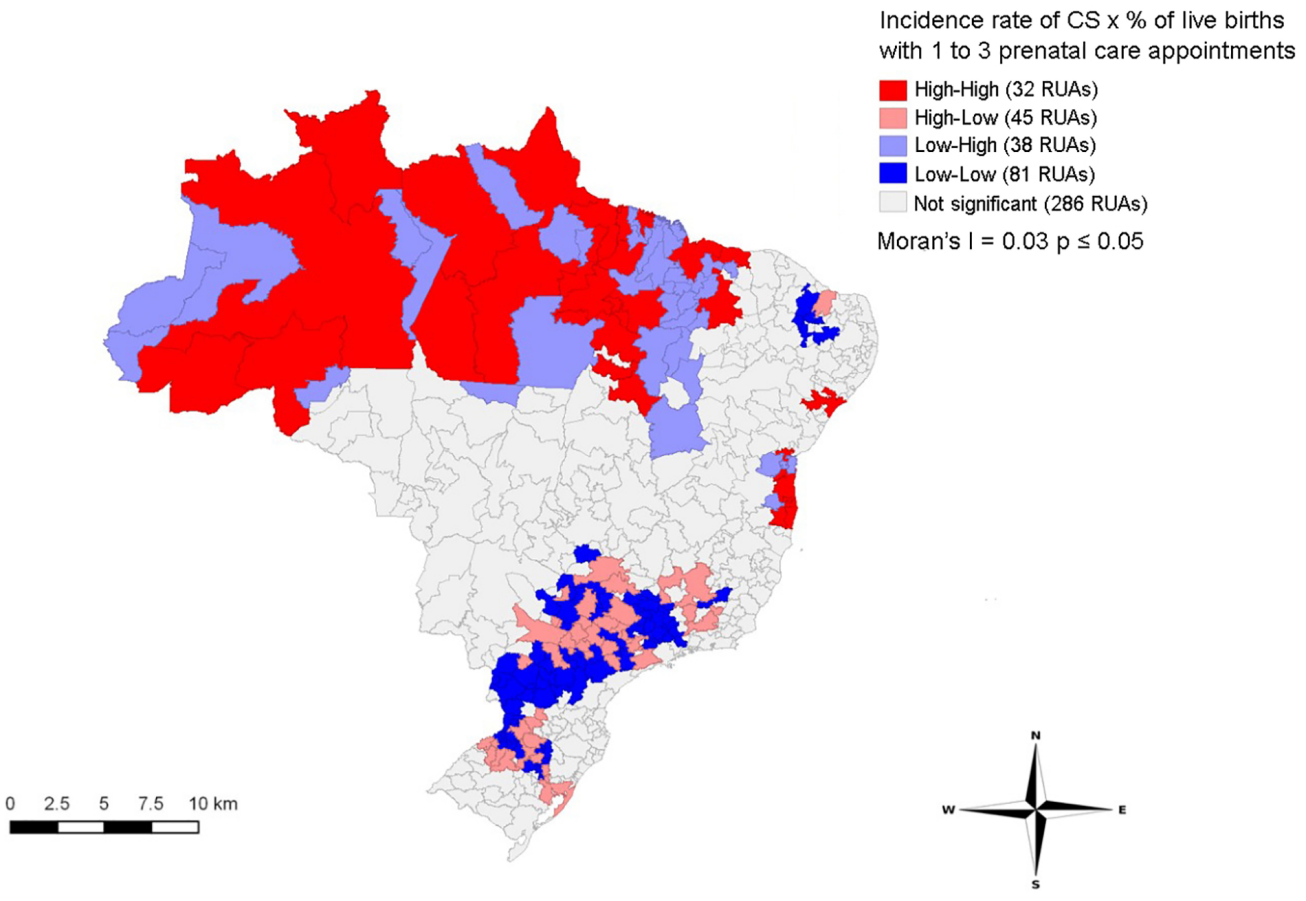
Distribution of LISA bivariate spatial correlation clusters of congenital syphilis incidence rate with the indicator prenatal care in immediate regions of urban articulation in Brazil, 2008–2018.

Figure 5 shows spatial correlations between the incidence rate of CS and percentage of live births with 1–3 prenatal care appointments (Moran’s *I* = 0.03, *p* ≤ 0.05). The LISA method indicated a direct correlation among clusters of the variables. RUAs in the North and part of Northeast (Maranhão, Piauí, Sergipe, and Bahia states) had larger clusters of CS and a high percentage of live births with 1 to 3 prenatal care appointments. On the other hand, RUAs in the South and Southeast presented clusters with high incidence of CS and low percentage of live births with 1–3 prenatal care appointments.

## Discussion

The historical series indicated an upward trend in the incidence of CS in Brazil, emphasizing the need for effective interventions. An inflection in 2013 was observed, with the highest APC between 2008 and 2013. However, despite a decrease in the growth curve in the following years, rates were still considered high, demonstrating lack of control of CS. Other American countries also presented an increased incidence (4), whereas other countries (e.g., China) reported a downward trend in the incidence of CS (18).

The spatial analysis highlighted CS as a public health problem in different Brazilian regions. RUAs covering Northeast coastal areas and Rio Grande do Sul, Rio de Janeiro, Mato Grosso do Sul states showed the highest incidence of CS. The central part of Northeast and areas in the Midwest presented RUAs with the lowest incidence rates of CS.

Teixeira et al. (19) suggested that the increased incidence of CS in the Rio Grande do Sul state was associated with increased testing and reporting of the disease, which reflects the implemented national public policies; however, the treatment of sexual partners was still a major healthcare problem. Another study in a municipality in the state of Rio de Janeiro showed that 42.4% of women were diagnosed with syphilis during delivery or curettage. Additionally, 68.6% of women were inadequately treated while 19.1% were not treated; only 12.2% of sexual partners were treated (20).

Cities close to international borders in Mato Grosso do Sul have an increased flow of people, goods, and drug trafficking, favoring the transmission of sexually transmitted infections. Also, the monitoring of indigenous peoples living in border regions is difficult (9). Tiago et al. (21) suggested that the magnitude of syphilis in indigenous peoples of Mato Grosso do Sul state may be hidden due to underreported cases, even with a high incidence rate. High-high patterns observed in coastal regions of the Northeast may be associated with the population context and great availability of healthcare services and maternity hospitals, resulting in increased notifications. In contrast, low-low patterns in the central region of the Northeast may be associated with the expansion of the Family Health Strategy program in recent years, suggesting effective prevention measures during prenatal care (22).

We observed a direct correlation between the incidence of CS and the percentage of individuals with inadequate water supply and sanitation. The North region showed more extensive clusters in areas with high incidence of CS and the percentage of individuals with inadequate water supply and sanitation. Thus, socioeconomic conditions may strongly influence the increase of CS in these regions.

Domingues and Leal (23) suggested that the increased incidence of CS in Brazil was influenced by social vulnerability. Studies also indicated associations between socioeconomic conditions and syphilis in the Northeast region (10, 24), with low purchasing power and poverty associated with increased cases of syphilis during pregnancy. Furthermore, studies carried out in a city in the state of Mato Grosso do Sul (9), in the state of Tocantins (25) and in Pará (26) showed a high incidence rates of syphilis in women with low education. In Pará, clusters for syphilis stand out in regions with a high flow of people and exposed to situations of social vulnerability. In the United States, researchers have identified the greatest increase in sexually transmitted infections, including syphilis, in metropolitan regions with greater population growth and in African-American populations (27). Thus, Brazilian women in poverty have difficulty accessing prenatal care and tests for syphilis (8).

An association was found between CS and the percentage of prenatal care appointments, an important indicator of access to prenatal care. The LISA method observed clusters of homogeneous areas with more pronounced spatial dependence, establishing priority areas. Brazilian regions present disparities in prenatal care, such as the number of appointments and services offered during appointments, especially testing for HIV and syphilis. The North region presented the lowest prenatal care coverage, whereas a high proportion of women from the North and Northeast reported three or few prenatal care appointments (28). Benzaken et al. (29) showed that 13.3% of pregnant women from Porto Velho (North region) did not have any prenatal care appointments, the highest percentage among Brazilian capitals. Moreover, poor areas in the North and Northeast have difficult access and low social conditions, leading to delayed implementation of healthcare programs and insufficient healthcare coverage (30).

RUAs in the South and Southeast regions showed clusters with high incidence of CS and low percentage of live births with 1–3 prenatal care appointments, suggesting increased access to prenatal care in these regions. However, the quality of prenatal care must be studied since clusters with a high incidence of CS were observed. The Moran’s *I* indicated a direct correlation between these variables, highlighting the importance of improving access and quality of prenatal care to control CS. Although pregnant women in the South region have more access to prenatal care (29), the treatment of pregnant women with syphilis and their partners is still challenging. Thus, cases of CS in this region are probably high due to lack or inadequate treatment (31, 32). Additionally, a study conducted in this same region showed that healthcare professionals presented insufficient knowledge about measures recommended by the Ministry of Health for preventing, treating, and controlling syphilis (33).

Lack of treatment during prenatal care was also observed in other countries. Kilball et al. (34) suggested that inadequate care, testing, and treatment during prenatal care were different between regions of the United States and contributed to increase syphilis cases during pregnancy and CS.

Reduced investments in public health, financial crisis, political instability, and freezing of funds in Brazil may have contributed to the increase in syphilis cases (35). Santos et al. (36) showed that several Brazilian cities lacked rapid tests for syphilis, reducing diagnosis, treatment, and control of syphilis in the population. Furthermore, some Brazilian regions should restructure the laboratory network to perform treponemal and nontreponemal tests (22).

The shortage of penicillin G due to lack of raw material in the world market, mainly in 2015, may have contributed to the increased incidence of CS (37). Also, Brazilian capitals presented a great variation in the availability of penicillin G benzathine in primary health care units (29) and difficulties in administering this medicine (36). Thus, policies should be developed to qualify and encourage healthcare professionals to administer penicillin G benzathine in basic health units, disseminate information about this medicine, and strengthen surveillance systems and healthcare networks in Brazil (33, 38).

Goals are set, worldwide, for dealing with syphilis (39). The Pan American Health Organization strengthens strategies for eliminating CS in countries (40). However, efforts have not been sufficient to control the disease and research addressing the issue needs to be continued. In Brazil, the Stork Network (41), the implementation of rapid tests (42), the compulsory notification of congenital syphilis (43), the agenda of strategic actions to reduction of syphilis (44) and the No Syphilis Project (45) are governmental measures instituted that strengthen the maternal and child area, which contribute in terms of increasing the detection and notification of the disease, but it has also not been sufficient to eliminate congenital syphilis in the country.

This study may have limitations due to the use of secondary data sources from Brazilian information systems and possible underreporting. The variable “pregnant women without prenatal consultation”, extracted according to the SINASC category, did not show statistical significance in the spatialization of data; thus, it was not included in the analysis. This result may have occurred due to the low number of cases or missing data, which we believe would not change the results. Although data regarding cases of CS were available only until 2018 in the SINAN, no major changes occurred in the context of the country. Because this is a study with aggregate data, there is a possibility of ecological fallacy. It is possible to carry out studies using spatial analysis focusing on regions of epidemiological interest, such as the clusters with high-high levels of incidence rate for CS pointed out in this research, with other independent variables to identify the association of new factors in the spatialization of CS.

Our results showed a growing trend of CS in Brazil, indicating that its eradication is still challenging for the country. The spatial analysis identified direct correlations between regions of clusters with high incidence of CS, access to prenatal care (1–3 appointments), and socioeconomic conditions (individuals with inadequate water supply and sanitation). Thus, inequalities in the Brazilian territory may be determinants of the spatial pattern of CS, while socioeconomic conditions and access to prenatal care may influence the incidence of CS in several Brazilian regions.

Therefore, our study may support public health policies to eradicate CS by indicating regions of greatest need for disease control. The importance of investments in socioeconomic policies must also be highlighted to reduce social inequality and improve access to prenatal care, contributing to managing maternal and child health in more susceptible areas.

## Data Availability

The raw data supporting the conclusions of this article will be made available by the authors, without undue reservation.
